# SALAD‐BAAR: A numerical risk score for hospital admission or emergency department presentation in ambulatory patients with cardiovascular disease

**DOI:** 10.1002/clc.23525

**Published:** 2021-02-02

**Authors:** Emeka C. Anyanwu, Rhys F. M. Chua, Stephanie A. Besser, Deyu Sun, James K. Liao, Corey E. Tabit

**Affiliations:** ^1^ Section of Cardiology, Department of Medicine University of Chicago Chicago Illinois USA

**Keywords:** hospital admission, risk prediction

## Abstract

**Background:**

While many interventions to reduce hospital admissions and emergency department (ED) visits for patients with cardiovascular disease have been developed, identifying ambulatory cardiac patients at high risk for admission can be challenging.

**Hypothesis:**

A computational model based on readily accessible clinical data can identify patients at risk for admission.

**Methods:**

Electronic health record (EHR) data from a tertiary referral center were used to generate decision tree and logistic regression models. International Classification of Disease (ICD) codes, labs, admissions, medications, vital signs, and socioenvironmental variables were used to model risk for ED presentation or hospital admission within 90 days following a cardiology clinic visit. Model training and testing were performed with a 70:30 data split. The final model was then prospectively validated.

**Results:**

A total of 9326 patients and 46 465 clinic visits were analyzed. A decision tree model using 75 patient characteristics achieved an area under the curve (AUC) of 0.75 and a logistic regression model achieved an AUC of 0.73. A simplified 9‐feature model based on logistic regression odds ratios achieved an AUC of 0.72. A further simplified numerical score assigning 1 or 2 points to each variable achieved an AUC of 0.66, specificity of 0.75, and sensitivity of 0.58. Prospectively, this final model maintained its predictive performance (AUC 0.63–0.60).

**Conclusion:**

Nine patient characteristics from routine EHR data can be used to inform a highly specific model for hospital admission or ED presentation in cardiac patients. This model can be simplified to a risk score that is easily calculated and retains predictive performance.

## 1 INTRODUCTION

Cardiovascular disease (CVD) is the leading cause of death globally as well as in the United States where the prevalence is 1 in 3.^1^ According to the American Heart Association, $1 out of every $6 in healthcare spending is spent on the management of CVD. This financial burden is growing and it will outpace capacity if costs of care are not reduced.^2^

Inpatient hospital care generally marks further decompensation in disease states and accounts for most of this financial burden. Of the nearly $200 billion in direct costs of CVD care in the United States, emergency department (ED), and inpatient care account for over $100 billion annually, more than the costs of office visits, home health, and medications combined.^3^ Estimates of the indirect costs of lost productivity were similarly high at $130 billion annually.

Several groups have designed interventions to reduce inpatient hospitalization with varying degrees of success.^4^, ^5^ Successful interventions are typically resource and labor intensive and require careful resource allocation to achieve cost effectiveness compared with routine practice. Therefore, identification of ambulatory patients at high risk for ED presentation or inpatient admission is of high importance. However, tools to predict ambulatory patients' risk of future admission are lacking. In this project, we developed a computational model that makes use of commonly available electronic health record (EHR) data to predict the risk of hospital admission or ED presentation in ambulatory cardiac patients.

## 2 METHODS

### 2.1 Study design and population

This retrospective cohort study included consecutive adult patient visits to the outpatient cardiology clinic at an urban academic tertiary referral center from 2013 to 2016. Patients were included in the cohort if they had been seen in the cardiology clinic at least twice in any given year. To avoid inclusion of patients for whom clinical management is atypical, patients with organ transplants, left ventricular assist devices, and home inotropes were excluded. The retrospectively analyzed clinic visits were randomized into a model training set (70% of visits) and a model testing set (30% of visits).

A separate dataset of patients seen in 2017 through 2018 was obtained after the model had been finalized and was used to perform prospective validation.

Data were collected in accordance with an approved Institutional Review Board (IRB) protocol under a general waiver of consent.

### 2.2 Study outcome

First, we determined whether each clinic visit was followed by a presentation to our ED or admission to our hospital for any reason within 90 days. We did not differentiate between hospital admission or ED presentation, because we felt both represented a notable escalation in level of care. Additionally, differentiating planned from unplanned admission or ED presentation is challenging in secondary analysis of EHR data. While the study population included patients with CVD who were treated in the cardiology clinic, all subsequent ED presentations or hospital admissions were included in the analysis regardless of presenting complaint or final diagnosis.

### 2.3 Model features

We collected data on each patient available in the EHR at the time of the clinic visit. Data included: demographic information, recent hospital admissions, vital signs, laboratory results, echocardiographic left ventricular ejection fraction, medications, prior diagnoses, and social vulnerability index variables related to the patient's home address.

International Classification of Disease (ICD) diagnosis codes (version 9 and 10) were categorized into major comorbidity groups according to a library defined in the Clinical Classifications Software developed by the Agency for Healthcare and Research Quality.^6^ A comorbidity diagnosis was considered present if a related ICD code was present in the patient's chart at the time of the clinic visit.

For each visit, the patient's median values for each continuous variable for the previous year were calculated. For each variable, the last year's median was subtracted from the most recent value to calculate a difference‐from‐median for each patient. The creation of these difference‐from‐median features served to assess for changes from each patient's normal values.

The social vulnerability index variables are a collection of census tract‐level data curated by the Geospatial Research, Analysis, and Services Program of the Centers for Disease Control and Prevention to reflect community resilience against stressors and natural disaster.^7^

### 2.4 Statistical analysis

Model features were assessed for collinearity using a Spearman rank correlation. When pairs of variables were found to be collinear, the more clinically relevant was kept in the analysis and the other was removed. Pairs that had a Spearman rank correlation of above 0.7 were removed; these included changes in lowest systolic blood pressure, change in lowest diastolic blood pressure, change in lowest pulse, and chronic obstructive pulmonary disease. When continuous variables (lab values, vital signs, social vulnerability index) were missing, they were imputed. For missing social vulnerability index variables, the mean was imputed. Calculated features (i.e., lab value and vital sign difference‐from‐median) that could not be calculated due to missing data were imputed to zero. Gender, comorbidities, clinical events, and medications were reported as frequencies and percentages (Table 1). Continuous variables were standardized to a mean of zero and a *SD* of one.

**TABLE 1:** Patient characteristics at the time of their outpatient cardiology clinic appointment

Patient characteristics	Datasets		
	Retrospective		Prospective
	Derivation *n* = 32 525	Testing *n* = 13 940	Validation *n* = 22 963
Age, median (IQR)	68 (57, 77)	68 (57, 77)	68 (57, 77)
Male % (*n*)	49 (15 822)	48 (6731)	48 (11 227)
Comorbidities % (*n*)			
Electrolyte disorders	27 (8718)	26 (3688)	36 (8172)
Anemia	32 (10 373)	32 (4449)	36 (8164)
Respiratory disease	27 (8868)	28 (3838)	31 (7134)
Hypertension	82 (26 610)	82 (11 398)	81 (18 624)
Diabetes	35 (11 348)	35 (4854)	35 (8106)
Atrial fibrillation	36 (11 594)	36 (4955)	38 (8782)
Heart failure	57 (18 648)	58 (8017)	58 (13 311)
Coronary disease	56 (18 285)	57 (7885)	55 (12 580)
Symptoms/clinical events % (*n*)			
Shortness of breath	42 (13 551)	42 (5803)	49 (11 354)
Blood loss	4 (1217)	4 (520)	5 (1112)
Previous 90‐day Admission	23 (7510)	23 (3217)	33 (7534)
Future 90‐day admission	19 (6323)	19 (2652)	25 (5838)
Medications % (*n*)			
Aspirin	18 (5958)	18 (2569)	18 (8527)
Nonaspirin antiplatelet	13 (4146)	12 (1651)	12 (5797)
Anticoagulant	23 (7434)	23 (3180)	25 (5835)
Diuretic	25 (8036)	25 (3458)	27 (6248)
Beta blocker	52 (16 999)	52 (7256)	56 (12 879)
Vitals, median (IQR)			
Systolic blood pressure (mmHg)	127 (115, 139)	127 (115, 139.4)	128 (117, 140)
Diastolic blood pressure (mmHg)	70 (64, 78)	70 (64, 78)	72.5 (66, 80)
Heart rate (BPM)	75 (67, 84)	75 (67.38, 84)	75 (67, 84)
Left ventricular ejection fraction (LVEF), median (IQR)	43.40 (25.20, 60)	42.70 (25.20, 60)	43.20 (29.52, 60)
Laboratories, median (IQR)			
Sodium (mEq/L)	139 (138, 141)	139.5 (138, 141)	140.5 (139, 142)
Potassium (mEq/L)	4.1 (3.9, 4.4)	4.1 (3.9, 4.4)	4.2 (3.9, 4.5)
Creatinine (mg/dl)	1.1 (0.9, 1.5)	1.1 (0.9, 1.5)	1.1 (0.9, 1.5)
Blood urea nitrogen (mg/dl)	19 (14, 27)	19 (14, 27)	19 (14, 27)
Albumin (g/dl)	3.95 (3.60, 4.20)	3.95 (3.60, 4.20)	4.00 (3.70, 4.20)
Low‐density lipoproteins (mg/dl)	89 (67, 120)	89 (67, 122)	92 (66, 128)
High‐density lipoproteins (md/dl)	49 (38.5, 61)	48 (38, 60)	46 (37, 59)
Triglycerides (mg/dl)	104 (76, 153)	104 (76, 153)	112.5 (81, 163.75)
Brain natriuretic peptide (pg/ml)	1976.5 (684, 4888.5)	2015.5 (718, 4852.5)	1435 (441.12, 4247.5)
Lactic acid (mEq/L)	1.60 (1.15, 2.15)	1.50 (1.15, 2.10)	1.62 (1.20, 2.30)
Lactate (mg/dl)	1.77 (1.20, 2.80)	1.90 (1.27, 2.80)	1.85 (1.20, 2.80)
Troponin (ng/ml)	0.03 (0.03, 0.06)	0.03 (0.03, 0.06)	0.03 (0.03, 0.05)
Erythrocyte sedimentation rate (mm/HR)	35 (20, 65)	33 (19.88, 60.62)	33 (18, 58.5)
Hemoglobin A1c (%)	6.90 (6.29, 8.05)	7.00 (6.25, 8.10)	6.95 (6.20, 8.15)
C‐reactive protein (mg/L)	13 (4, 46)	12.25 (3, 40.5)	9 (3, 28)

The retrospective dataset was divided randomly into a derivation set and testing set in a ratio of 70:30. The derivation set was used to train the model, while the testing set was kept aside to assess performance of each model iteration.

The full model feature set was used to train a gradient‐boosted decision tree model (XGBoost). Feature importance analysis of the decision tree model was used to eliminate less important features. In the XGBoost model, “gain” was used to define feature importance. Gain represents the improvement in model accuracy attributable to branches where the feature is present. Area under the curve (AUC) score was used to evaluate XGBoost model performance.

The top selected features were used to train a multivariate logistic regression model. The odds ratios resulting from the logistic regression were used to develop a simplified numerical prediction score by rounding the odds ratios down to the nearest whole integer with a total possible score of 10. The highest possible score was the sum of the rounded integers.

The discriminatory power (c‐statistic) of various numerical prediction score cutoffs were assessed with the final cutoff selected to maximize the c‐statistic. The final numerical prediction score was evaluated for sensitivity and specificity.

Data processing and analyses were performed with freely available open‐source software: R (version 3.5.1), Python (version 3.7.4), Sci‐kit Learn (version 0.21.3), Pandas (version 0.25.1), Numpy (version 1.16.5), StatsModels (version 0.10.1), and XGBoost (version 0.90).

## 3 RESULTS

During the study period a total of 46 465 clinic visits for 9326 unique patients met inclusion criteria and were included in the retrospective analysis. 75 patient characteristics were evaluated for importance as described above. 9096 clinic visits were followed by an ED presentation or hospital admission within 90 days for an event rate of 20%. 4191 clinic visits were followed by an ED or hospital admission within 30 days for an event rate of 10%.

Patient characteristics at the time of their clinic visits are given in Table 1. Median age at the time of the clinic appointment was 68 years, and men and women were near‐equally represented. Comorbidities and medications at the time of clinic appointment are also given in Table 1.

The XGBoost model was the most accurate with a receiver operating characteristic AUC of 0.75, compared with the logistic regression AUC of 0.73. Feature importance analysis of the XGBoost model yielded 10 features that were more important than others (Figure S1). SVI variables did not contribute to the AUC score of XGBoost; the model without SVI only affected the score by 0.001.

The top 10 features were: Shortness of breath, Aspirin usage, Lytes (electrolyte disorders), Anticoagulant drugs, Diuretics (loop diuretics), Blood loss, 90‐day Admission (previous admission in the preceding 90 days), Anemia, Respiratory Disease, and 30‐day admission (previous admission in the preceding 30 days). Previous 30‐day admission was removed from this list to leave a final list of the top 9 model features for the following reasons: (a) It was collinear with admission in the previous 90 days (Spearman rank coefficient of 0.64) and (b) it had the least relative importance of the top 10 features. We refer to these top 9 features acronymically as the SALAD‐BAAR Score.

**FIGURE 1:**
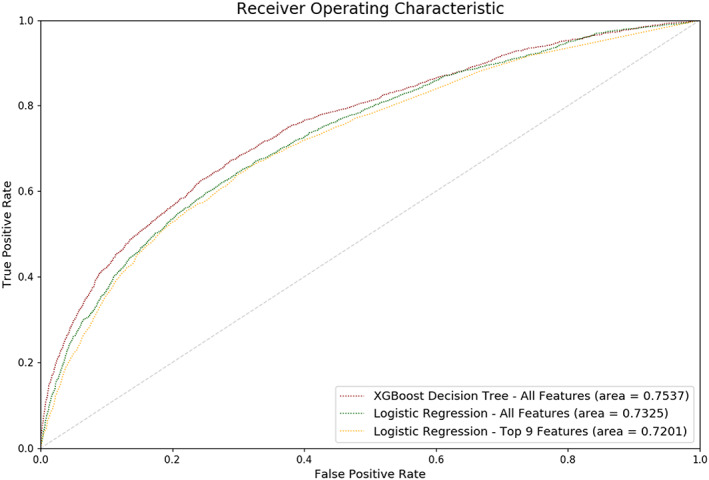
Receiver operating characteristic curves (ROC) for the XGBoost and logistic regression models

**FIGURE 2:**
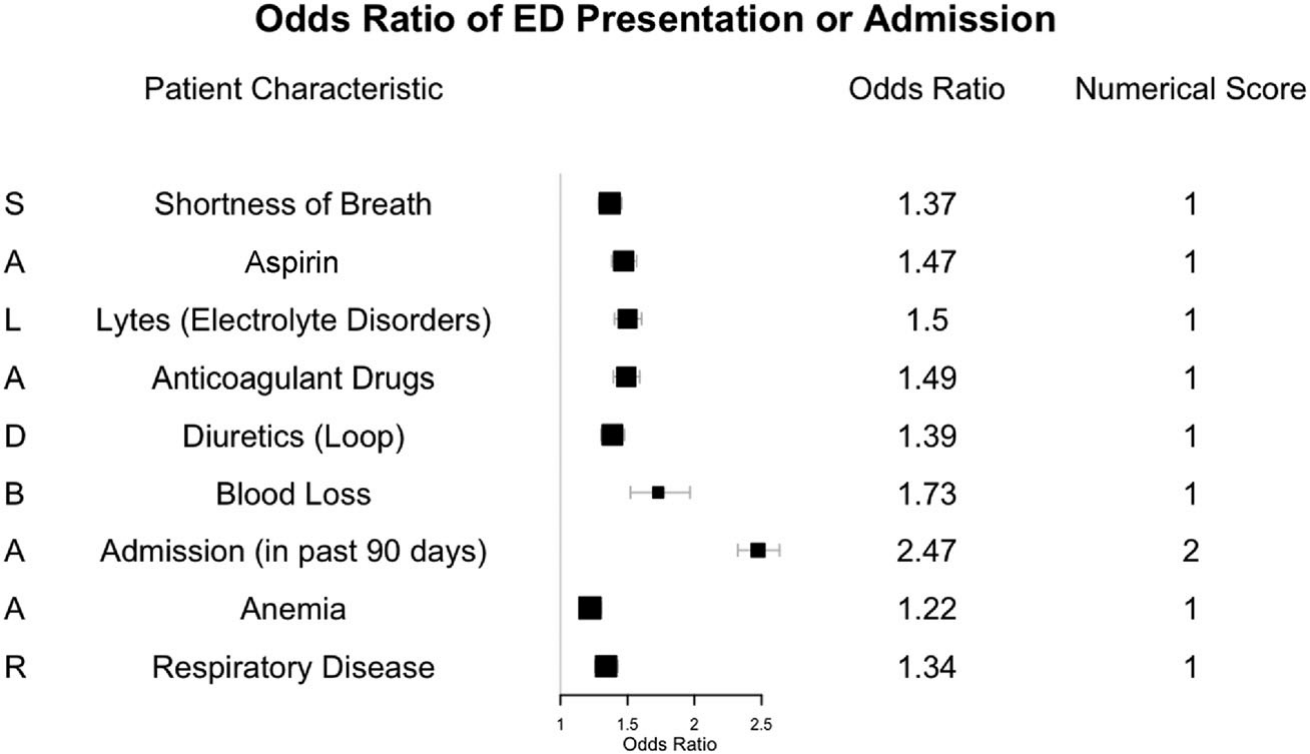
Odds ratios for the logistic regression model and their resulting numerical scores. ED, emergency department

The top 9 features were used to train a multivariate logistic regression model without the other features. The receiver‐operating characteristic curves for the XGBoost and logistic regression models are shown in Figure 1. The odds ratios from the limited feature logistic regression model were rounded down to the nearest integer to form our simplified numerical score with a total possible score of 10 (Figure 2). This simplified numerical score model achieved a sensitivity of 0.58, specificity of 0.72, and an AUC of 0.66. The odds ratios and their resulting scores are shown in Figure 2.

**FIGURE 3:**
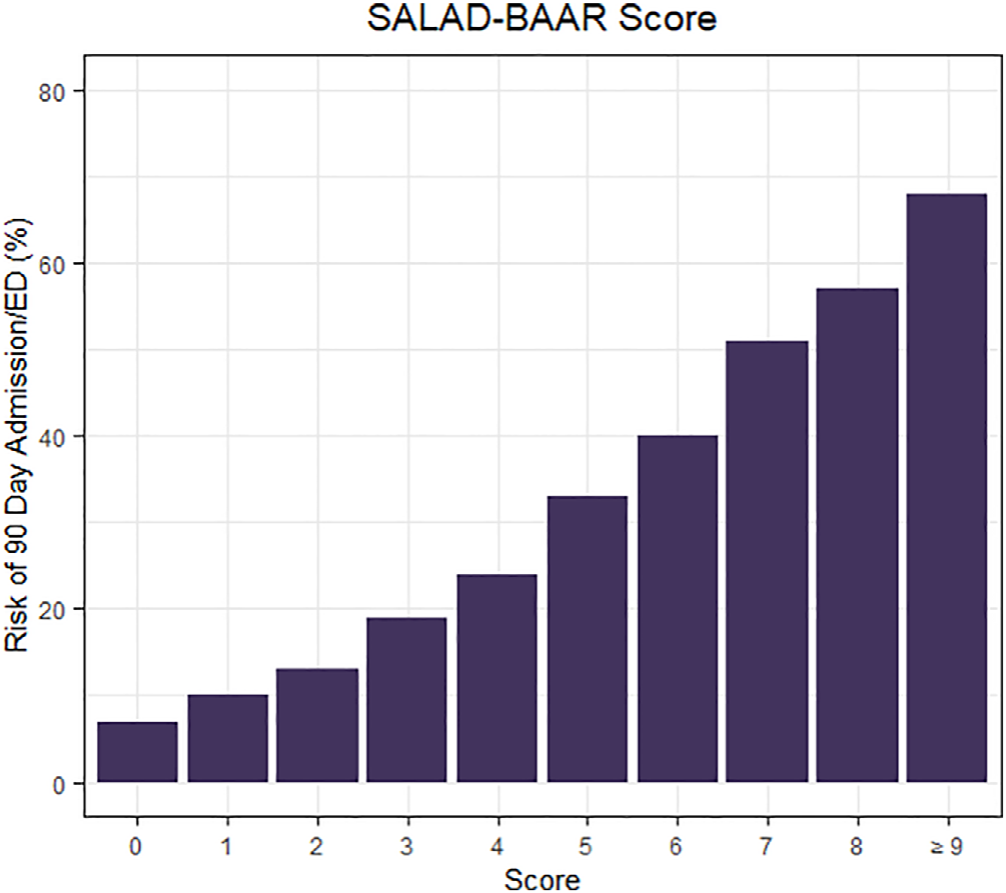
Risk of 90‐day admission or emergency department (ED) visit versus SALAD‐BAAR score

The risk of 90‐day admission or ED visit was then calculated for increasing values of the SALAD‐BAAR Score. The risk of 90‐day admission or ED visit increases as the SALAD‐BAAR Score increases as shown in Figure 3. Stratification by age and gender produced very similar results for ROC (Figure S2(A–D)), coefficients (Figure S3(A–D)), and admission rates (Figure S4(A–D)).

Finally, prospective validation of the model using data from 2017 to 2018 resulted in sensitivity of 0.58, specificity of 0.68, and AUC of 0.63. In the validation dataset, 49% of the 6367 patients were also present in the derivation (training and test) dataset. This accounted for 54% of the 22 963 encounters. Analysis of the numerical score was repeated including only the patients who were not present in the derivation dataset. This repeat analysis resulted in a sensitivity of 0.49, specificity of 0.72, and an AUC of 0.60. The AUC, sensitivity, specificity of all models and cohorts are given in Table 2.

**TABLE 2:** Performance metrics for all constructed models

Model	Sensitivity	Specificity	AUC
Retrospective			
XGBoost (all features)	0.24	0.97	0.75
Logistic regression (all features)	0.18	0.97	0.73
Logistic regression (9 features)	0.17	0.97	0.72
Numerical score (9 features)	0.58	0.75	0.66
Prospective			
Numerical score (9 features)	0.58	0.68	0.63
Numerical score (9 features, repeat patients excluded)	0.49	0.72	0.60

## 4 DISCUSSION

In our study of 46 465 cardiology clinic visits with 9326 patients at a tertiary academic center, we developed the SALAD‐BAAR score that predicts the risk of 90‐day hospital admission or ED presentation. Our model was prospectively validated using a separate dataset of 22 963 encounters and 6367 unique patients.

Our outcome variable of hospital admission in the 90 days following a cardiology clinic appointment occurred at a rate of 20%, consistent with the highly morbid nature of CVD in our patient population. Our algorithm was able to predict the likelihood of hospital admission or ED presentation in the 90 days following cardiology clinic visit with sensitivity of 0.58, specificity of 0.68, and AUC of 0.63. These metrics compare favorably to other large cohort numerical prediction scores, such as those used to predict cardioembolic stroke in the setting of atrial fibrillation^8^, ^9^ or the likelihood of potentially avoidable 30‐day hospital readmission in medical patients.^10^

Amongst our prediction variables, we found the use of diuretics, aspirin, and anticoagulant medications were significant markers of increased risk in our patient population. Given that these medications are most often prescribed to manage advanced symptoms (such as heart failure) or reduce the risk of major adverse cardiac events (such myocardial infarction or stroke), it follows that they signal increased risk for acute hospital presentation.

Electrolyte abnormalities also were found to be an important predictor of acute presentation. Hyponatremia is known to be a marker of dysregulation of the renin‐angiotensin system in acute and chronic CVD and has been associated with increased mortality in several studies.^11^, ^12^, ^13^ In addition, abnormalities of potassium and magnesium are frequent side effects of the high doses of diuretics used to manage symptoms in severe heart failure.

Likewise, patients with severe CVD are more likely to have blood loss and anemia, which can result from acute blood loss related to surgery or procedures but may also be secondary to chronic causes. Severe aortic stenosis has been associated with acquired von Willebrand deficiency from increased shear forces that can lead to chronic blood loss.^14^ Finally, anemia is present in one‐third of patients with heart failure often secondary to the effects of chronic inflammation, renal insufficiency, and nutritional deficiency.^15^

The diagnosis of respiratory disease and the clinical symptom of shortness of breath were also identified as high‐powered predictors in our model. Comorbid respiratory disease or symptoms of shortness of breath may reflect chronic secondary remodeling or damage to the pulmonary system due to severe heart disease such as in cor pulmonale. Shortness of breath is also a common clinical manifestation of acute cardiac conditions, such as acute heart failure or myocardial infarction.

Unsurprisingly, prior hospital admission or ED presentation within 90 days was the strongest predictor of future presentation. This finding reflects similar data from other studies, including the HOSPITAL score, a risk stratification tool to predict the risk of hospital readmission following discharge.^10^, ^16^

The results of this study must be assessed in the context of their limitations. First, the data analyzed are from a single hospital, and thus, we are unable to account for admissions that occurred at other local hospitals. However, CMS and self‐reported data analyzed as part of a previous hospital admissions study at our institution noted that patients were only admitted to other hospitals 5% of the time.^4^ While said study specifically investigated heart failure patients, we do not have reason to believe that this would be different for all of our center's cardiology patients.

Second, our study relies on secondary analysis of EHR data. In the diagnosis codes especially, this information should be interpreted with caution. Prior literature has highlighted concerns with accuracy of EHR diagnosis codes.^17^ Potential reasons for this include provider frustration with data entry and time pressure during order signing. In order to reduce variability introduced by user‐entered ICD codes we made use of comorbidity group classifications defined by the Agency for Healthcare and Research Quality.^6^ These groupings place diagnosis codes into broad clinically relevant categories instead of relying on the granular accuracy of user entries. Future research including clinical note review may improve the accuracy of our model.

Additionally, while the derivation and initial testing of our model were based on retrospective data, our model's observed performance was consistent when applied to a hold‐out prospective validation dataset. While we find these results promising, an external validation is required before this algorithm should be applied more broadly. Specifically, external validation in patient populations disparate from our own would enhance the generalizability of our model.

## 5 CONCLUSION

The SALAD‐BAAR Score is a simple tool to predict future 90‐day ED presentation or hospital admission in ambulatory cardiac patients. The score performed well when prospectively validated with single‐center data. This score, the first of its kind for ambulatory cardiac patients, has the potential to identify patients who may benefit from interventions to reduce their risk of hospital admission.

## CONFLICT OF INTEREST

The authors declare no potential conflict of interest.

## Supporting information


**Figure S1** XGBoost model feature importance analysis. The horizontal value represents the improvement in model accuracy attributable to branches where the feature is present. The top 9 features used for the development of the score are highlighted in purple. SVI – social vulnerability index.
**Figure S2** Receiver operating characteristic curves (ROC) for the XGBoost and logistic regression models stratified by gender and age: (a) male, (b) female, (c) under 65 years old, (d) over 65 years old.
**Figure S3** Odds ratios for the logistic regression model and their resulting numerical scores stratified by gender and age: (a) male, (b) female, (c) under 65 years old, (d) over 65 years old.
**Figure S4** Risk of 90‐day admission or ED visit versus SALAD‐BAAR Score stratified by gender and age: (a) male, (b) female, (c) under 65 years old, (d) over 65 years old.

## Data Availability

Data are available by request to the corresponding author subject to approval by the IRB at the University of Chicago.
