# Supplementary material for: SALAD‐BAAR: A numerical risk score for hospital admission or emergency department presentation in ambulatory patients with cardiovascular disease
Source: Clin Cardiol. 2021 Feb 2;44(2):193–9. doi: 10.1002/clc.23525 (PMC7852175; doi:10.1002/clc.23525)
Supplement: Supplementary file 1 — Figure S1 XGBoost model feature importance analysis. The horizontal value represents the improvement in model accuracy attributable to branches where the feature is present. The top 9 features used for the development of the score are highlighted in purple. SVI – social vulnerability index. Figure S2 Receiver operating characteristic curves (ROC) for the XGBoost and logistic regression models stratified by gender and age: (a) male, (b) female, (c) under 65 years old, (d) over 65 years old. Figure S3 Odds ratios for the logistic regression model and their resulting numerical scores stratified by gender and age: (a) male, (b) female, (c) under 65 years old, (d) over 65 years old. Figure S4 Risk of 90‐day admission or ED visit versus SALAD‐BAAR Score stratified by gender and age: (a) male, (b) female, (c) under 65 years old, (d) over 65 years old. [file CLC-44-193-s001.docx]

**FIGURE S1**


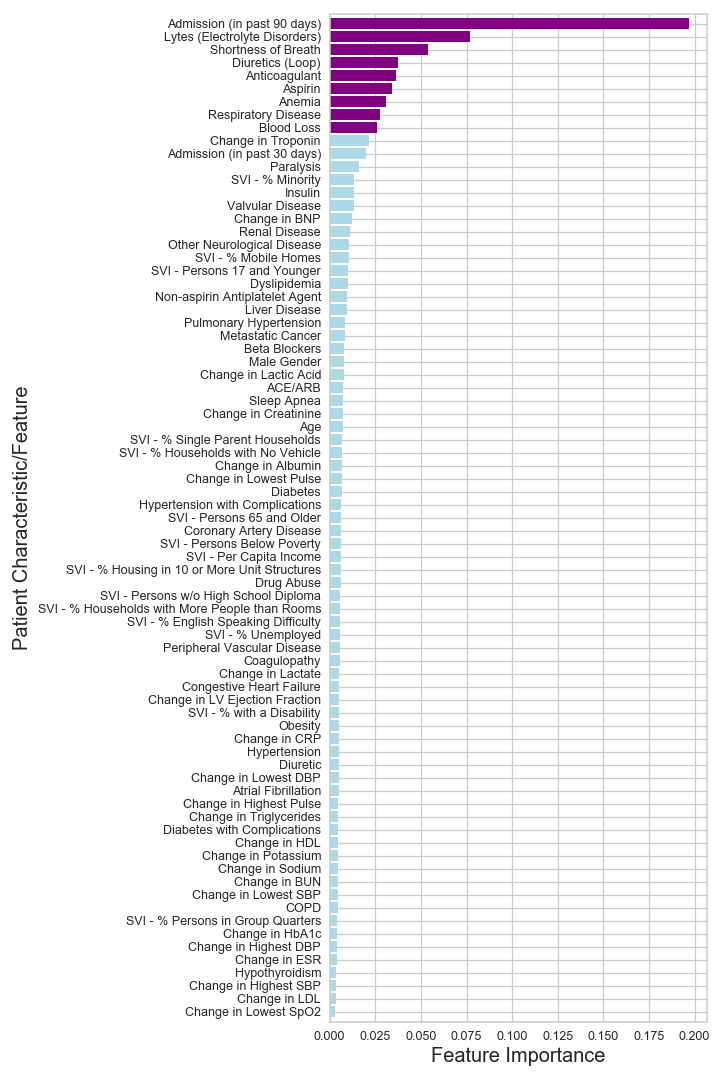


**Figure S2a - Male**

**
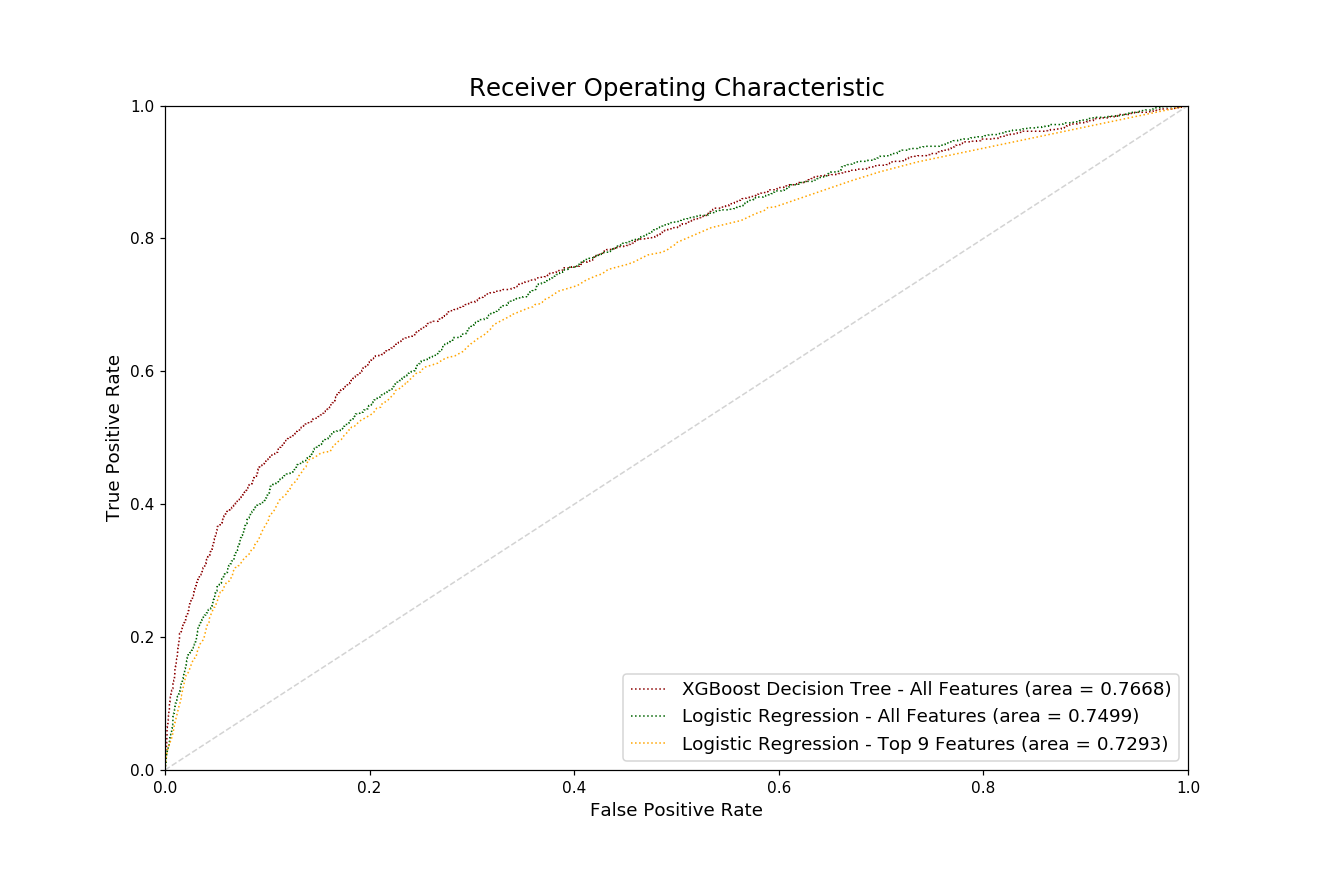
**

**Figure S2b – Female**

**
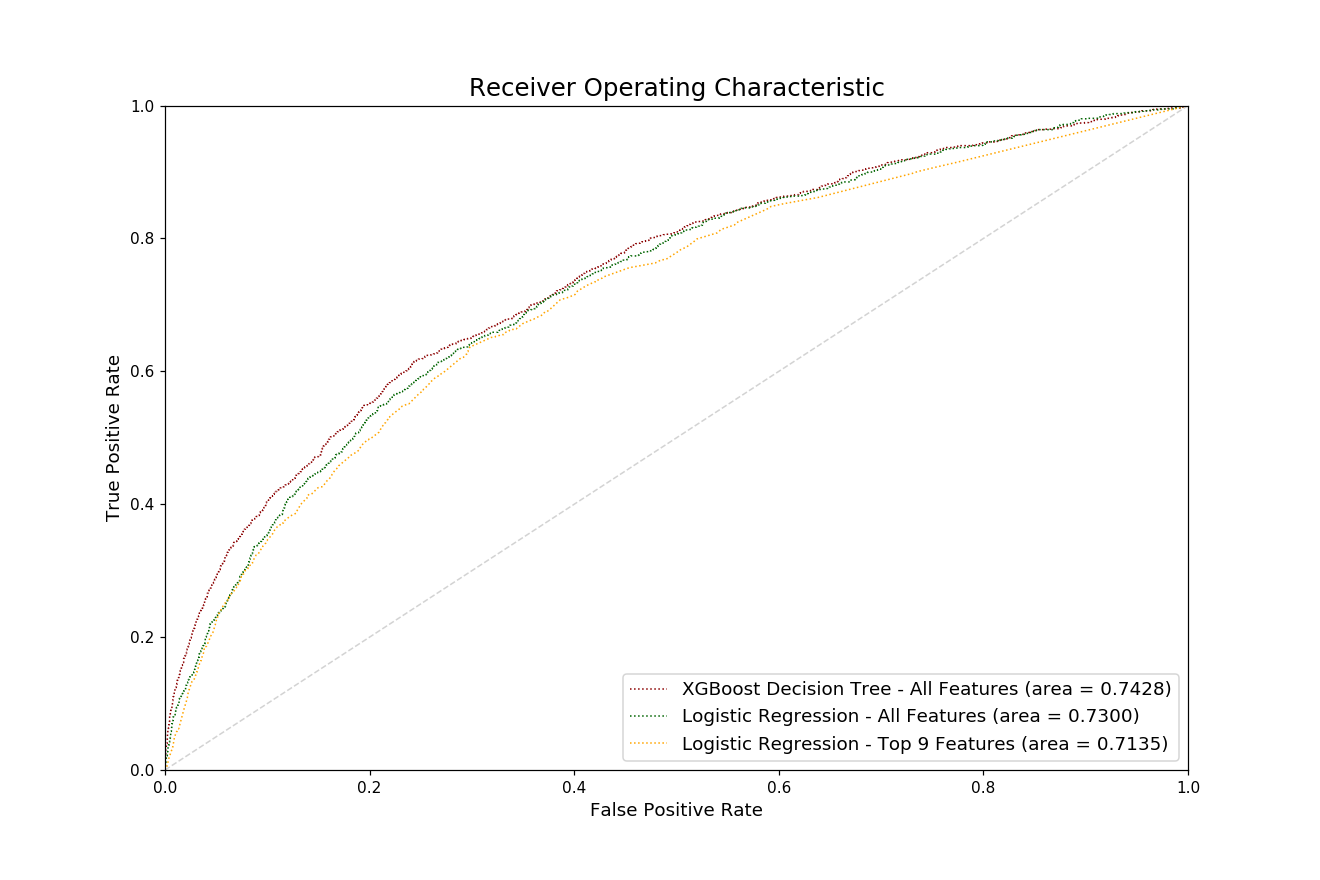
**

**Figure S2c – Under 65**

**
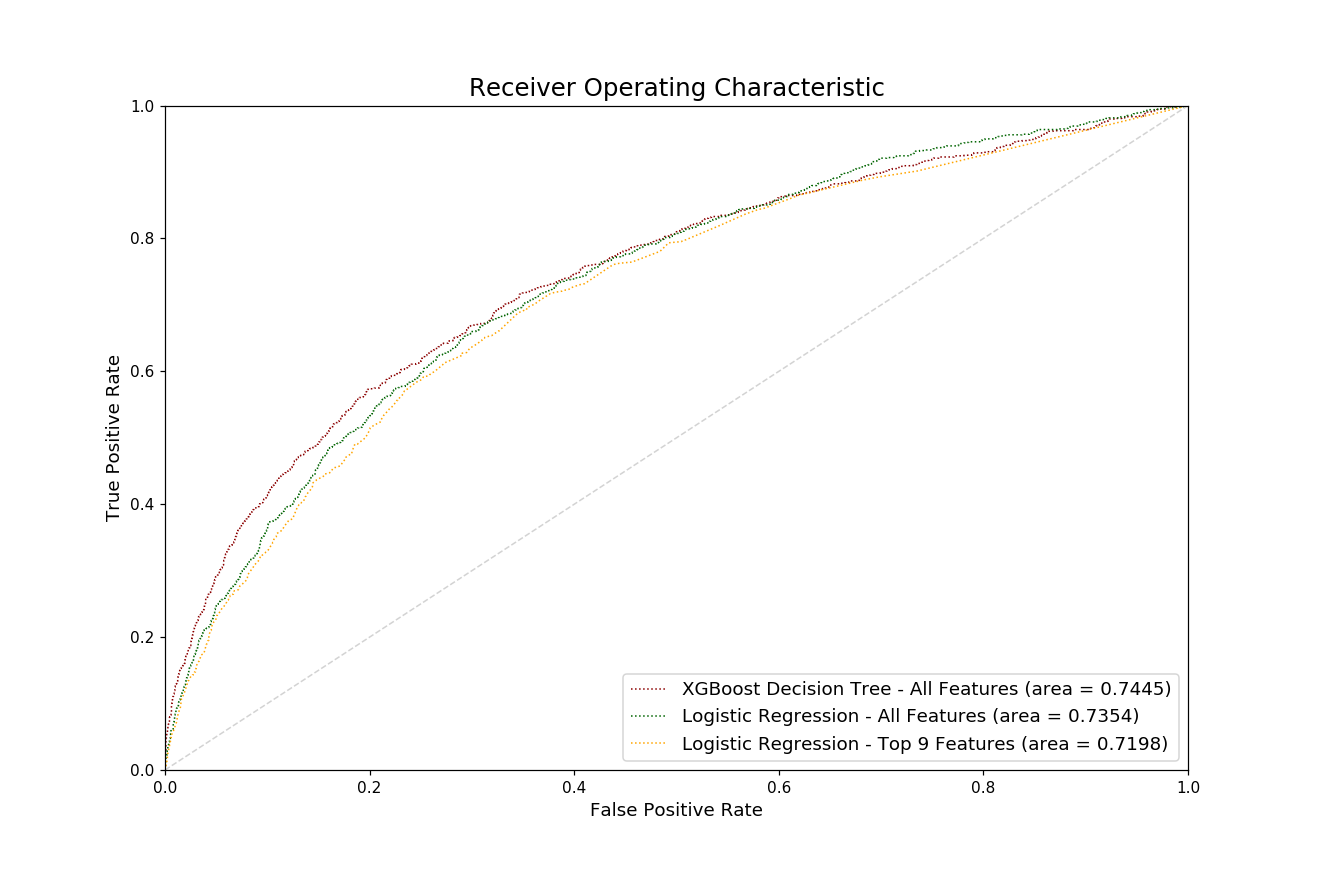
**

**Figure S2d – Over 65**

**
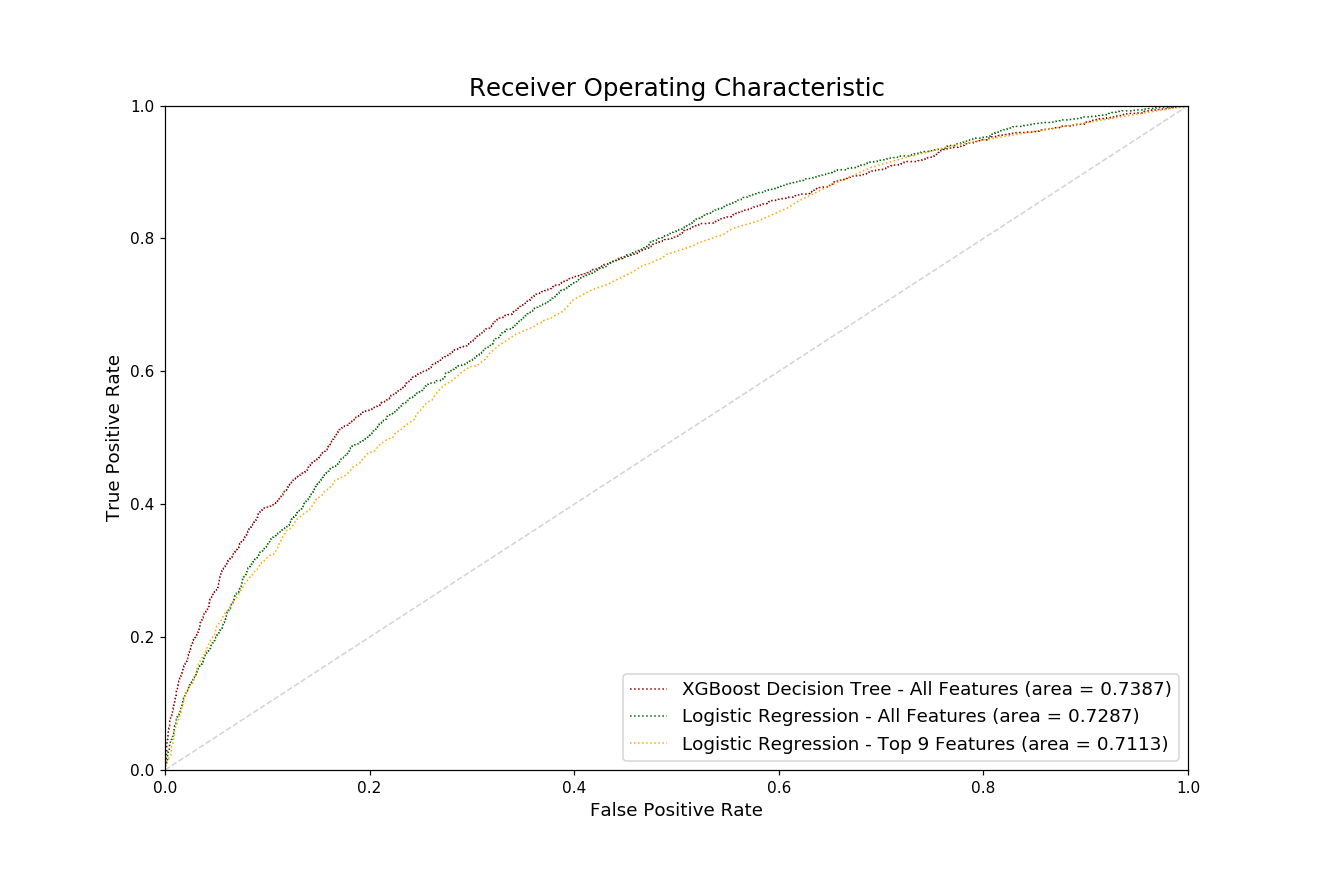
**

**Figure S3a - Male
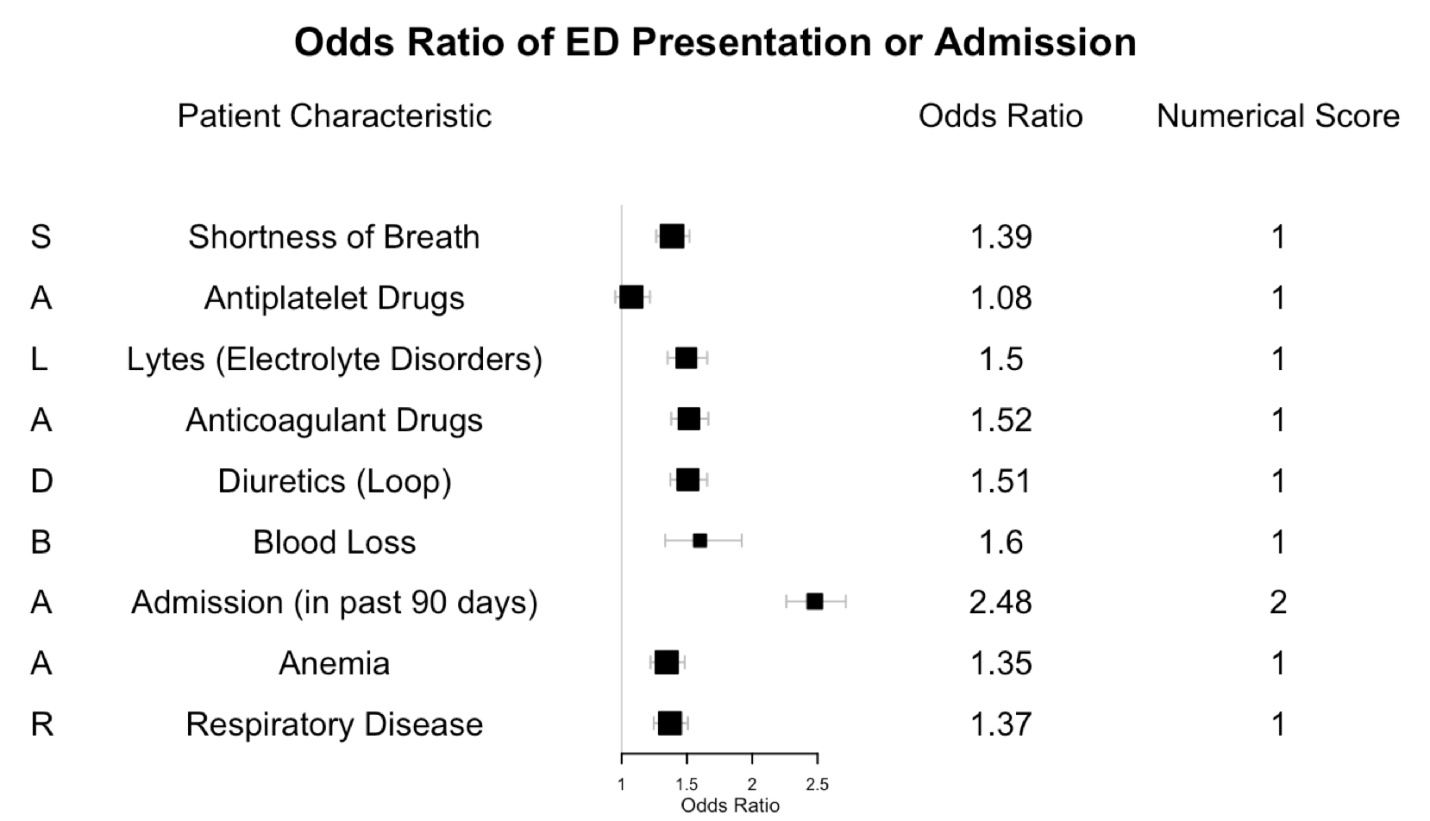
**

**Figure S3b – Female**

**
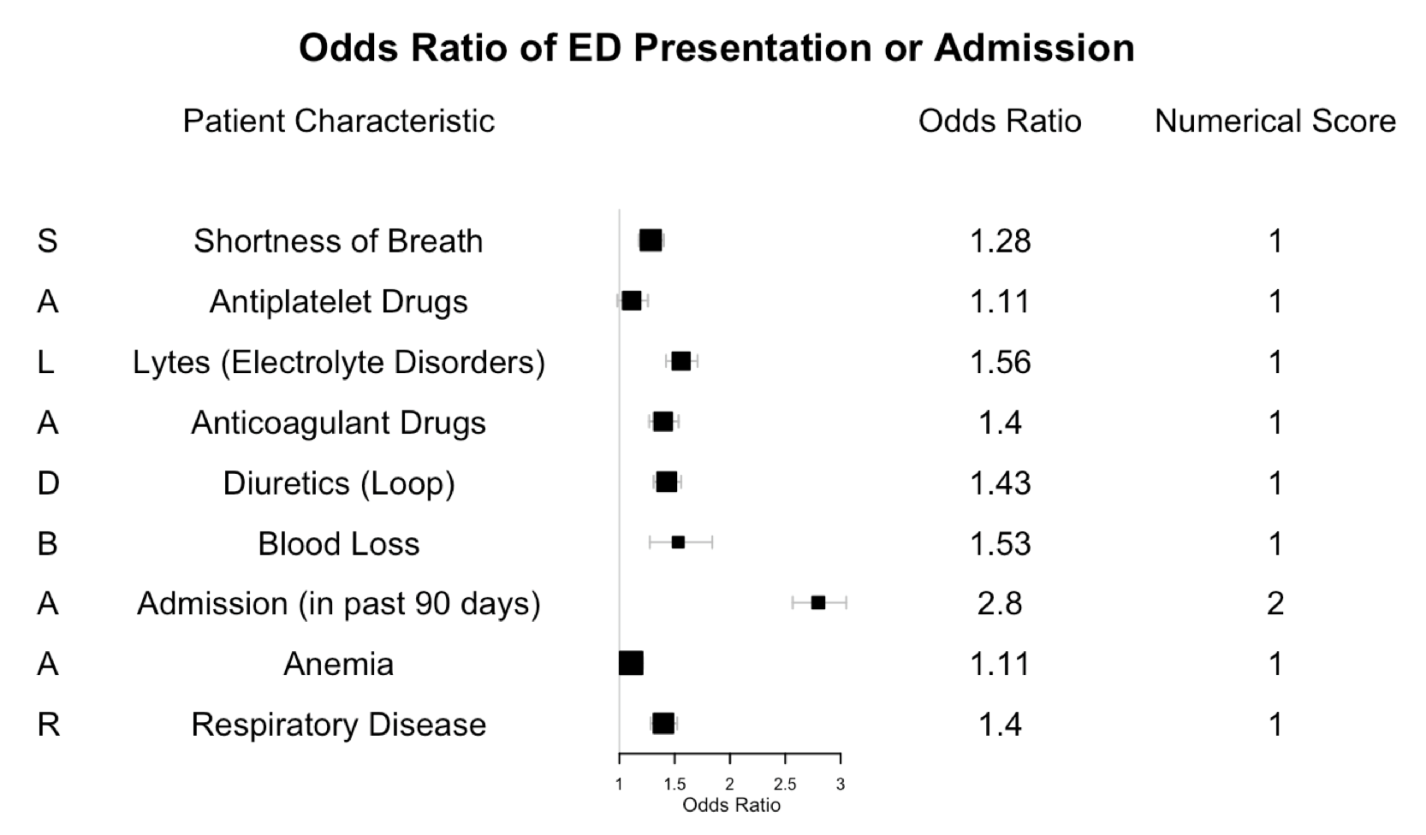
**

**Figure S3c – Under 65**

**
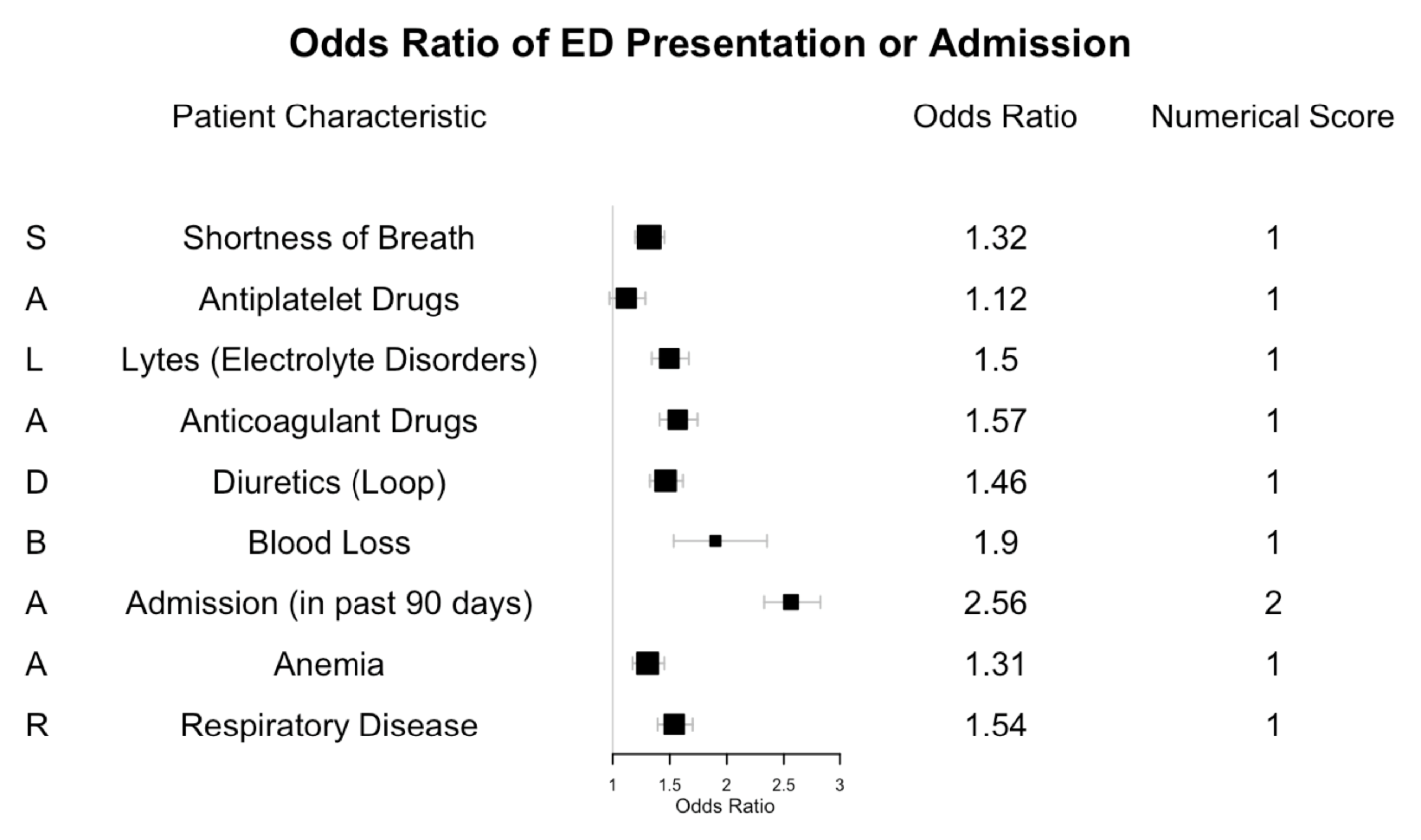
**

**Figure S3d – Over 65**

**
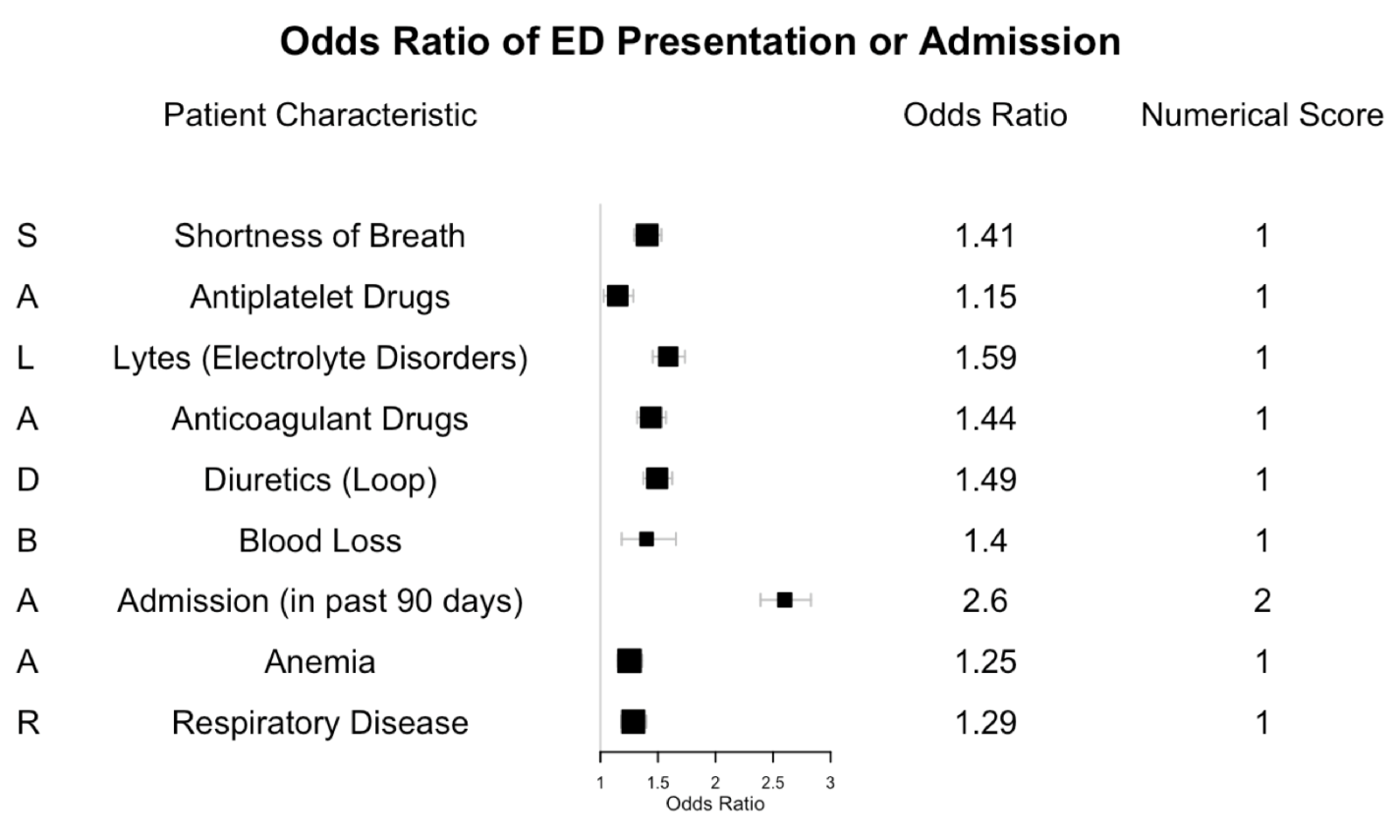
**

**Figure S4a – Male**

**
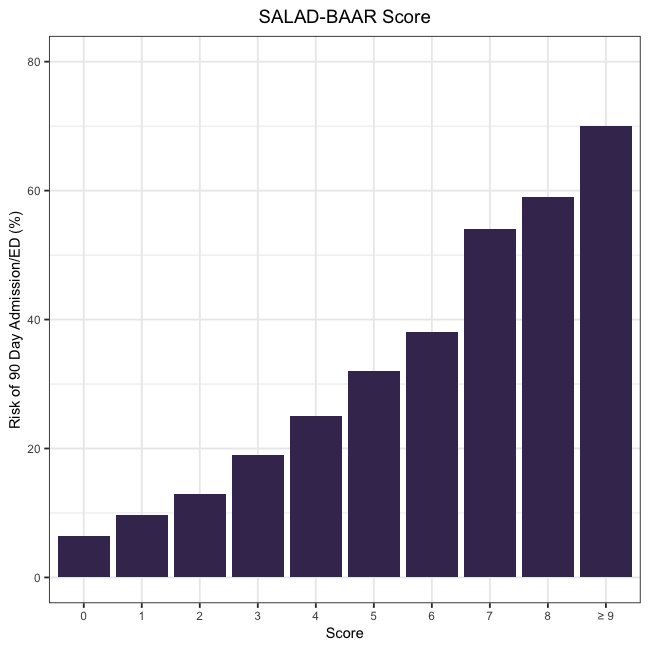
**

**Figure S4b – Female**

**
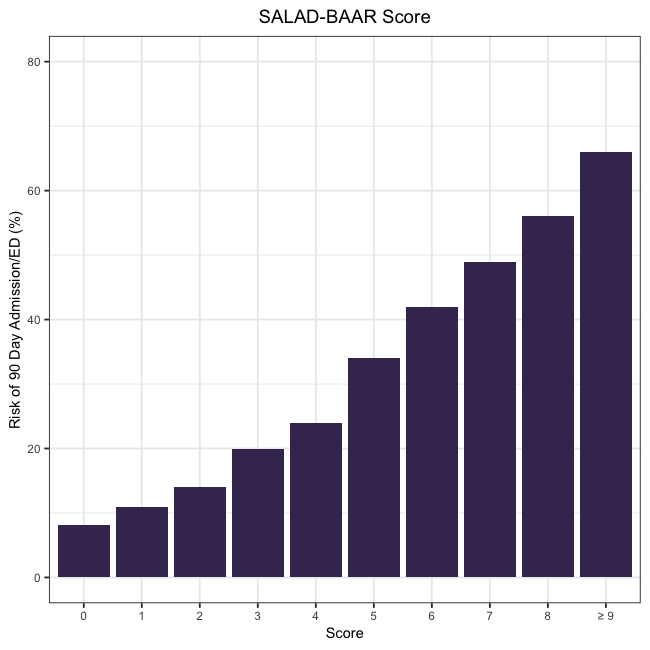
**

**Figure S4c – Under 65**

**
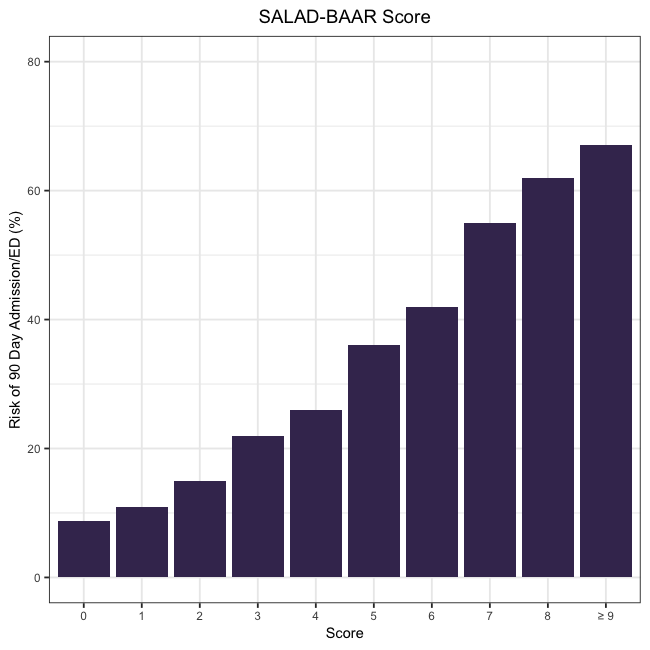
**

**Figure S4d – Over 65**

**
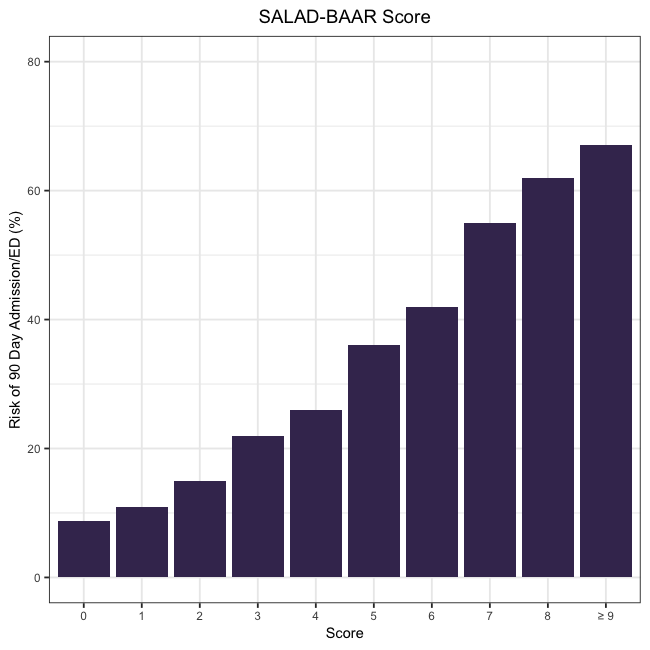
**
